# Challenges of Clustering Multimodal Clinical Data: Review of Applications in Asthma Subtyping

**DOI:** 10.2196/16452

**Published:** 2020-05-28

**Authors:** Elsie Horne, Holly Tibble, Aziz Sheikh, Athanasios Tsanas

**Affiliations:** 1 Usher Institute, Edinburgh Medical School University of Edinburgh Edinburgh United Kingdom

**Keywords:** asthma, cluster analysis, data mining, machine learning, unsupervised machine learning

## Abstract

**Background:**

In the current era of personalized medicine, there is increasing interest in understanding the heterogeneity in disease populations. Cluster analysis is a method commonly used to identify subtypes in heterogeneous disease populations. The clinical data used in such applications are typically multimodal, which can make the application of traditional cluster analysis methods challenging.

**Objective:**

This study aimed to review the research literature on the application of clustering multimodal clinical data to identify asthma subtypes. We assessed common problems and shortcomings in the application of cluster analysis methods in determining asthma subtypes, such that they can be brought to the attention of the research community and avoided in future studies.

**Methods:**

We searched PubMed and Scopus bibliographic databases with terms related to cluster analysis and asthma to identify studies that applied dissimilarity-based cluster analysis methods. We recorded the analytic methods used in each study at each step of the cluster analysis process.

**Results:**

Our literature search identified 63 studies that applied cluster analysis to multimodal clinical data to identify asthma subtypes. The features fed into the cluster algorithms were of a mixed type in 47 (75%) studies and continuous in 12 (19%), and the feature type was unclear in the remaining 4 (6%) studies. A total of 23 (37%) studies used hierarchical clustering with Ward linkage, and 22 (35%) studies used k-means clustering. Of these 45 studies, 39 had mixed-type features, but only 5 specified dissimilarity measures that could handle mixed-type features. A further 9 (14%) studies used a preclustering step to create small clusters to feed on a hierarchical method. The original sample sizes in these 9 studies ranged from 84 to 349. The remaining studies used hierarchical clustering with other linkages (n=3), medoid-based methods (n=3), spectral clustering (n=1), and multiple kernel k-means clustering (n=1), and in 1 study, the methods were unclear. Of 63 studies, 54 (86%) explained the methods used to determine the number of clusters, 24 (38%) studies tested the quality of their cluster solution, and 11 (17%) studies tested the stability of their solution. Reporting of the cluster analysis was generally poor in terms of the methods employed and their justification.

**Conclusions:**

This review highlights common issues in the application of cluster analysis to multimodal clinical data to identify asthma subtypes. Some of these issues were related to the multimodal nature of the data, but many were more general issues in the application of cluster analysis. Although cluster analysis may be a useful tool for investigating disease subtypes, we recommend that future studies carefully consider the implications of clustering multimodal data, the cluster analysis process itself, and the reporting of methods to facilitate replication and interpretation of findings.

## Introduction

### Background

There is mounting evidence to suggest that some disease labels are in fact *umbrella terms*, which encompass distinct disease subtypes with different underlying mechanisms and clinical symptom manifestations [1-3]. This has encouraged the investigation into heterogeneity within disease populations, which has received considerable interest across diverse domains of medicine [4-6]. There are numerous motivations for better understanding heterogeneity within disease populations, from the development of targeted therapeutics [6] to the delivery of more personalized care in clinical practice [7].

It is now understood that asthma is one such umbrella term used to encompass multiple diverse underlying disease symptoms and pathophysiology [7]. Asthma is a common chronic condition characterized by reversible airway obstruction. The Global Burden of Disease Study 2017 estimated the global prevalence of asthma (both symptomatic and asymptomatic) to be 273 million [8]. This study estimated that in 2017, there were 43 million new cases of asthma and 495,000 deaths attributed to asthma [9]. Attempts to categorize asthma into distinct disease subtypes date back to the 1940s [10] and are ongoing. However, the methods for discovering these underlying categories have shifted from observing clinical patterns to using data-driven approaches such as *cluster analysis* [11].

Cluster analysis is a statistical technique used to identify subgroups in data based on multiple variables (for convenience, herein, we have used the term *features*). It is an *unsupervised* statistical learning method, and the correct number of underlying clusters is typically unknown *a priori* [12]. The technique has found increasing use in recent years because of the practical unmet clinical need to identify subtypes of disease and stratify patients to improve health care delivery. This has been made feasible by the increasing availability of clinical datasets and the development of statistical software packages facilitating the application of algorithmic methods.

Clinical datasets are often *multimodal*; for the purposes of this paper, we defined a multimodal dataset as a dataset that includes features from different sources, measured on different scales. For completeness and to avoid ambiguity, we clarified that the term multimodal has a different meaning in statistical literature (ie, features with multiple modes in terms of its distribution); the use of the term in this study is aligned with clinical literature (having features from different sources). Popular methods of cluster analysis such as k-means and hierarchical clustering with the Ward method have been developed for continuous features measured on a common scale. In practice, however, many of these techniques are frequently applied to multimodal clinical datasets comprising different feature types measured on different scales, conditions that violate some of the underlying principles and assumptions made by algorithmic methods [13]. Although steps can be taken to prepare multimodal clinical data for cluster analysis [13], the results of a previous review suggest that these steps are rarely taken in practice [11]. This previous review focused on the clinical findings of the studies and touched only briefly on the challenges of clustering multimodal data specifically.

### Objectives

This review aimed to comprehensively explore whether studies applying cluster analysis to multimodal clinical data to subtype asthma are using appropriate clustering methodologies. The contribution of this study is to make recommendations for the robust application of cluster analysis to multimodal clinical data. We believed this would be of interest to the ever-growing number of asthma researchers engaging or planning to engage in disease subtyping, as well as to the wider community of researchers applying cluster techniques for the purpose of disease subtyping.

## Methods

### Eligibility Criteria and Search Strategy

This review is reported following the Preferred Reporting Items for Systematic Reviews and Meta-Analyses (PRISMA) guidelines. Multimedia Appendix 1 shows the completed PRISMA checklist.

We sought to identify studies that applied cluster analysis to multimodal clinical data with the aim of identifying subtypes of asthma. One researcher (EH) searched PubMed and Scopus databases (search queries are provided in Textbox 1) to retrieve studies focusing on patients diagnosed with asthma, which included the term *cluster analysis* or *clustering*. Our search was restricted to studies published between January 1, 2008, and May 23, 2019, as Haldar et al’s study [14] is widely acknowledged to be the first to apply cluster analysis to identify subtypes of asthma. Our search excluded comment articles, editorials, letters, reviews, and meta-analyses. We excluded articles that were not written in English.

We excluded nonrelevant studies by first screening the abstracts, then referring to the full text where necessary. We excluded studies in which (1) none of the aims or objectives were to identify subtypes of asthma (studies looking exclusively at, eg, childhood wheeze were excluded); (2) the data were not multimodal (ie, were measured from a common source and on a common scale); and (3) none of the features were considered clinical (eg, studies concerned only with -omics data). Finally, we excluded studies that used latent class analysis or mixture models to group their data to narrow the scope of this review to methods that cluster samples based on pairwise dissimilarities. The use of latent class analysis to distinguish asthma phenotypes has been reviewed previously by Howard et al [15].

**Search query to identify studies to include in this review.**

- The following query was inserted in PubMed on May 23, 2019:

English[Language] AND (“2008/01/01”[Date - Publication] : “2019/05/23”[Date - Publication]) AND (“cluster analysis”[Text Word] OR “clustering*”[Text Word]) AND “asthma*”[Text Word] NOT (comment[Publication Type] OR editorial[Publication Type] OR letter[Publication Type] OR review[Publication Type] OR meta-analysis[Publication Type])

- The following query was inserted in Scopus on May 23, 2019:

PUBYEAR > 2007 AND (TITLE-ABS-KEY ( “cluster analysis” ) OR TITLE-ABS-KEY(“clustering*”)) AND TITLE-ABS-KEY (“asthma*”) AND SRCTYPE (“j”) AND DOCTYPE (“ar”) AND LANGUAGE (“English”)

### Data Extraction

In total, 2 researchers (EH and HT) independently extracted information from the full text and supplementary material of each study. Information was extracted following the steps outlined in the following *Cluster Analysis Steps* section. The data dictionary, which provides details of all items extracted, is presented in Multimedia Appendix 2.

### Cluster Analysis Steps

**Figure 1:**
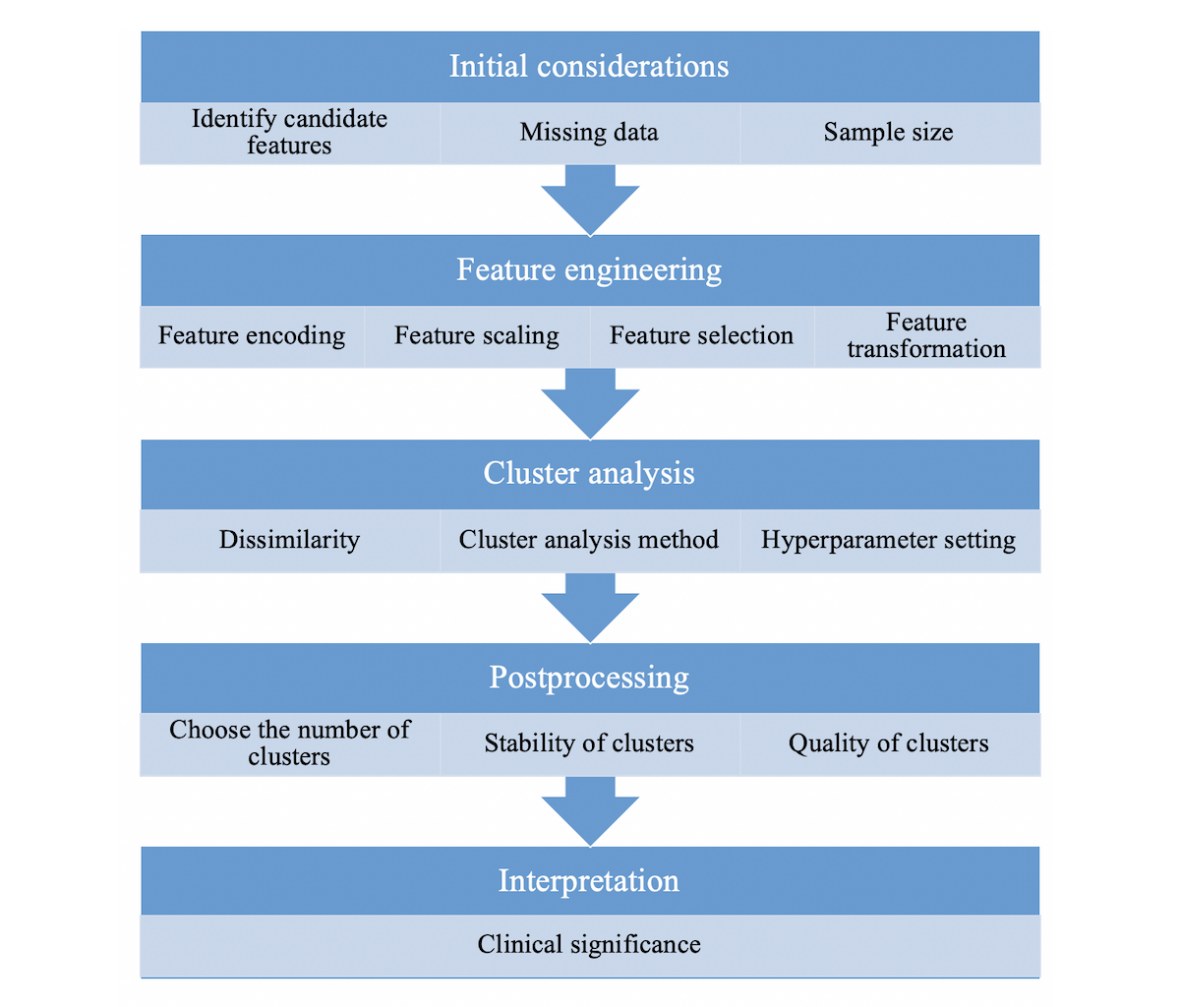
Schematic of the typical cluster analysis steps.

To provide context for this review, we outlined the key steps in the application of cluster analysis to multimodal clinical data. Figure 1 summarizes the steps in the order in which they generally occur, but as with most analytic processes, this depends on the context, and the process may be somewhat iterative.

#### Initial Considerations

##### Identify Candidate Features

The first step is to identify the set of features of interest, which we referred to as *candidate features*. These may be identified based on previous studies or clinical input using domain expertise. In some cases, all the candidate features may be used in the cluster analysis (we referred to the features used in cluster analysis as *cluster features*). In other cases, formal feature selection processes may be applied to the candidate features to identify the cluster features, as covered in the *Feature Selection* section.

##### Missing Data

Most common cluster analysis methods use *complete case analysis* (ie, the cluster features have no missing entries, which, in practice, might be achieved by removing samples for which any cluster feature entry is missing). However, it may be more data efficient to develop a strategy to work around missing entries instead of discarding samples. Missing values may be handled through the calculation of dissimilarities, as described by Hastie et al [16]. Alternatively, missing data could be imputed, or for categorical features, a missing category could be introduced.

##### Sample Size

Despite the widespread use of cluster analysis, at present, there is no consensus regarding the minimum sample size required to ensure stable and meaningful clustering. Dolnicar et al [17] suggested that 70 samples per cluster feature is adequate, based on the findings of their simulation study. Small sample sizes may obscure the true clustering by causing the user to pick the wrong number of clusters (see the *Choosing the Number of Clusters* section) or by producing solutions that are neither reproducible nor stable (see the *Stability* and *Quality* subsections).

#### Feature Engineering

##### Feature Types

The features that we may want to use in a clustering algorithm often come from multimodal clinical data. Hence, they may be of different types (eg, continuous, nominal, ordinal, binary, etc) and are likely to be measured on different scales (eg, kilogram for mass, years for age). Most dissimilarity measures and clustering algorithms assume that the features are of the same type and are measured on a common scale. These requirements can be addressed using *feature encoding* and *feature scaling*.

##### Feature Encoding

When dealing with categorical features, it is vital to consider how these are encoded (nominal, ordinal, or binary), as this determines how they are treated in the calculation of dissimilarities and in the clustering algorithm. A common approach is to encode ordinal features as integers and to encode nominal features as dummy binary features [18].

##### Feature Scaling

Feature scaling may be used to address 3 issues related to continuous features. The first is that continuous features may be measured in different units and should therefore be rescaled to bring them onto a common scale before calculating dissimilarities. The second is that continuous features measured in the same units may have different variances. In some cases, the differences in variance may be useful for clustering, but in others, these may obscure the true underlying cluster structure in the data. In the latter case, the continuous features should be rescaled. Common approaches to these 2 issues are to standardize features to have 0 mean and unit variance (referred to as *z-scores*) or to use range normalization techniques, for example, to scale each feature so that it is in the interval of 0 to 1.

The third issue is that the features may not follow the desired probability distribution properties for further analysis (eg, having Gaussian-distributed features). This issue needs to be considered when statistical methods make distributional assumptions. Although few dissimilarity-based clustering methods make distributional assumptions, several methods involve the calculation of cluster means (eg, k-means, hierarchical clustering with the Ward linkage). The mean is a poor choice of summary statistic for a feature that is skewed (or a feature with multiple modes), so a power transformation may be advantageous as a preprocessing step when using such clustering methods.

When dealing with mixed-type data, it may be necessary to scale the categorical features to avoid assigning categorical features greater weight over continuous features or vice versa. This issue is discussed in detail in the context of dissimilarity measures by Hennig and Liao [13].

##### Dimensionality Reduction

There are generally 2 motivations for reducing the dimensionality of a dataset before applying cluster analysis. First, as previously mentioned in the *Sample Size* subsection, datasets with a high feature to sample ratio may not produce stable cluster results. Second, the cluster structure may only be apparent using a subset of the information available in the data. Using all available information may introduce noise, which could obscure the true underlying cluster structure [19]. There are 2 approaches to dimensionality reduction: *feature selection* and *feature transformation*.

##### Feature Selection

Feature selection involves selecting a subset of the available features for use in cluster analysis. Herein, we have referred to the features selected for the cluster analysis as *cluster features*.

##### Feature Transformation

Feature transformation involves combining original features to create new features. Generally, a subset of these new features is selected for inclusion in the analysis. It is beyond the scope of this review to provide in-depth details on the methods of feature transformation (also known as *feature extraction*); we referred to van der Maaten et al’s [20] work for a comprehensive review. Here, we briefly outlined *principal component analysis* (PCA), which is the most commonly used method for linear data projection. PCA may be applied to *p* continuous, correlated features to extract *m<p* continuous, and uncorrelated features (known as *principal components*), each being a linear function of the original cluster features [21]. Related methods include factor analysis for continuous data, *multiple correspondence analysis* (MCA) for categorical data [22], and multiple factor analysis for mixed-type data [23].

#### Cluster Analysis

##### Dissimilarity Measures

Model-free clustering methods rely on a *dissimilarity measure* to quantify how dissimilar 2 samples are from one another. Dissimilarity may also be referred to as a *distance measure* if it satisfies the triangle inequality. The most widely used dissimilarity measure is the squared Euclidean distance (henceforth referred to as *Euclidean distance*), which is intended for use with continuous features. A dissimilarity measure that can handle both categorical and continuous features is the Gower distance [24].

##### Cluster Analysis Methods

There are many different methods of cluster analysis (eg, k-means, hierarchical clustering with the Ward linkage, spectral clustering), and each method may be implemented using different algorithms. A comprehensive overview of the wide range of clustering methods can be found elsewhere [25].

#### Postprocessing

##### Choosing the Number of Clusters

A key challenge in cluster analysis is choosing the number of clusters to present in the final solution, which is typically unknown *a priori*. Often, researchers use their preferred clustering methods, running them for 2 to *k* clusters (where *k* is an integer number indicating the number of clusters) and then have a strategy to determine *k*.

Providing a detailed commentary on these strategies is beyond the scope of this review. An overview of strategies for choosing *k* is provided by Everitt et al [23]. Graphical techniques include dendrograms (when using hierarchical clustering methods) and silhouette plots [26]. An alternative approach is to choose the number of clusters that gives the most stable solution [27]. In practice, a key determinant in choosing the number of clusters is often the clinical interpretation of the solutions.

We highlighted the possibility that there might not be meaningful clustering of the data to form groups, and thus, the entire dataset is treated as 1 cluster. This may reflect the lack of statistical power (sufficiently large sample size) to determine clusters or that the investigated problem using that dataset is not amenable to clustering using the available sample size and features. Some statistics used for choosing k, such as the Gap statistic [28], can be calculated for k=1. However, statistics that require the calculation of between cluster differences or distances, such as the silhouette statistic, are not defined for k=1 [26].

##### Stability

Assessing the quality of a clustering solution produced using any cluster algorithm is challenging. Unlike supervised learning setups, there is no *ground truth* against which one can formally test their findings. However, there are several ways in which one can assess the integrity of their findings.

Most importantly, it is crucial to assess the *stability* of the resulting clusters. A definition of *cluster stability,* given by von Luxburg [27], is whether clustering different datasets sampled from the same underlying joint distribution will result in producing the same clusters. There are several ways in which this may be assessed in practice (eg, by comparing the cluster results of a dataset that has been randomly split into 2 or more subsets, and each subset is independently fed into the cluster algorithm).

##### Quality

Beyond stability, there are numerous steps one may take to ensure the integrity of their cluster analysis findings, for example, repeating the analysis in a different cohort or at a different time point, or altering the encoding of a feature. These steps are often referred to as reproducibility testing. However, we avoided this term because it implies that we seek the exact same results, which we do not feel is reasonable in all scenarios. To extract this information from the studies in this review, 2 reviewers independently extracted details of postprocessing methods, which we felt assessed the quality of the cluster results, but did not come under stability. In our schematic and results, we referred to these methods as testing the quality of the cluster results.

## Results

### Literature Search Outcomes

**Figure 2:**
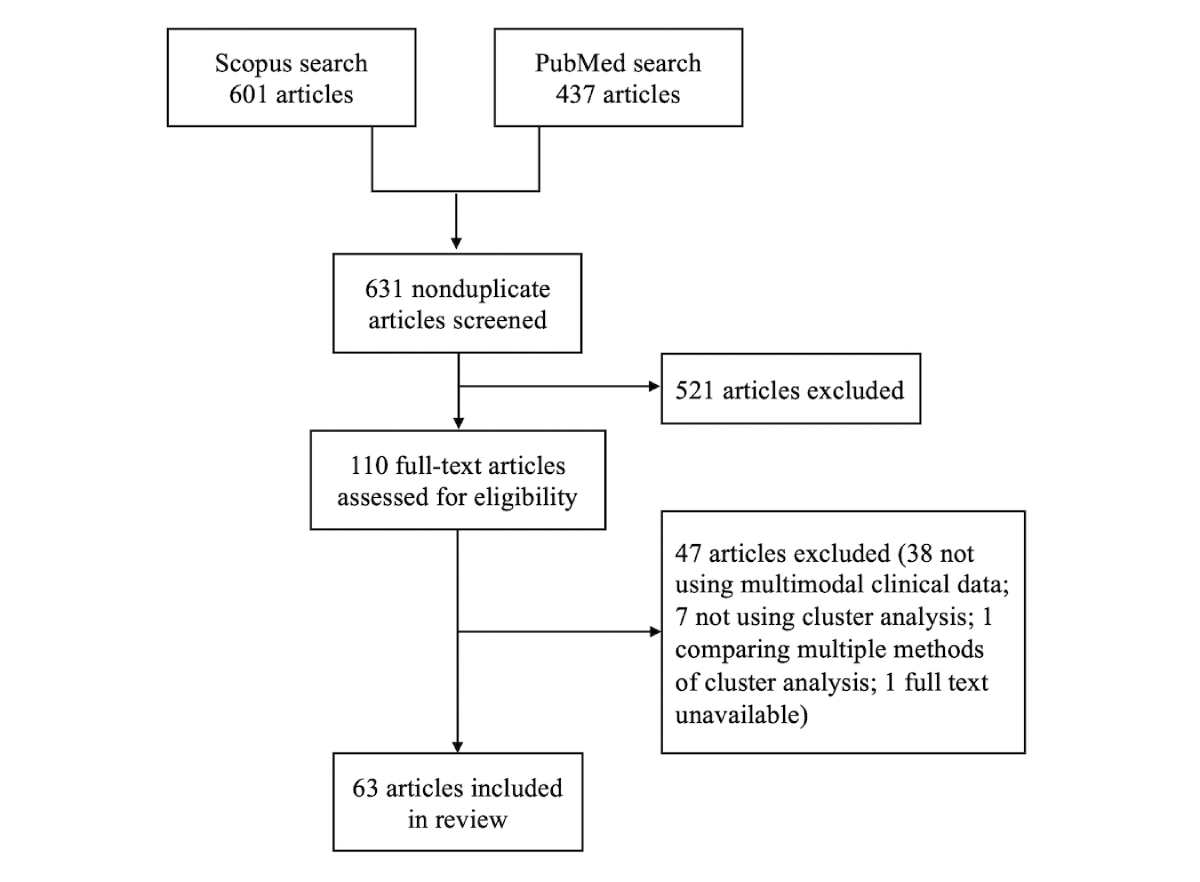
Flow of studies into review.

We identified 63 studies that used cluster analysis to identify subtypes of asthma using multimodal clinical data (Figure 2). One of the excluded articles satisfied our inclusion criteria but investigated 85 combinations of cluster analysis steps in a hierarchical cluster analysis of 383 children with asthma [29]. We excluded this study from our review as including all 85 combinations of methods was deemed infeasible. For the 2 studies in which cluster analysis was carried out in multiple populations [14,28], we included only the analysis of the larger population. The characteristics of each study are presented in Multimedia Appendix 3.

### Initial Considerations

#### Identifying Candidate Features

A total of 42 (67%) studies identified candidate features based on previous studies or clinical input (relevance to asthma subtypes, avoiding clinical redundancy, and easily measured in clinical practice). The numbers used in each method are summarized in Table 1.

#### Missing Data

A total of 42 (67%) studies detailed their methods for dealing with missing data; the methods used are shown in Table 1. The most common method was to carry out a complete case analysis by excluding all patients with any missing cluster feature entries (35% of studies).

**Table 1:** Initial considerations across the asthma studies we have included in this review (N=63).

Method		Values, n (%)^a^
**Identifying candidate features**		
	Clinical intuition and understanding	33 (52)
	Avoid clinical redundancy	15 (24)
	Previous studies	15 (24)
	Easily measured in clinical practice	8 (13)
**Missing data**		
	Complete case analysis	22 (35)
	Features with >x%^b^ missing values removed	14 (22)
	Imputed	11 (17)
	Patients with >x%^b^ missing values removed	5 (8)
	No missing data present	2 (3)
	Clustering methods handle missing data	1 (2)

^a^One study may use multiple methods; some studies may use no methods.

^b^x>0.

#### Sample Size

**Figure 3:**
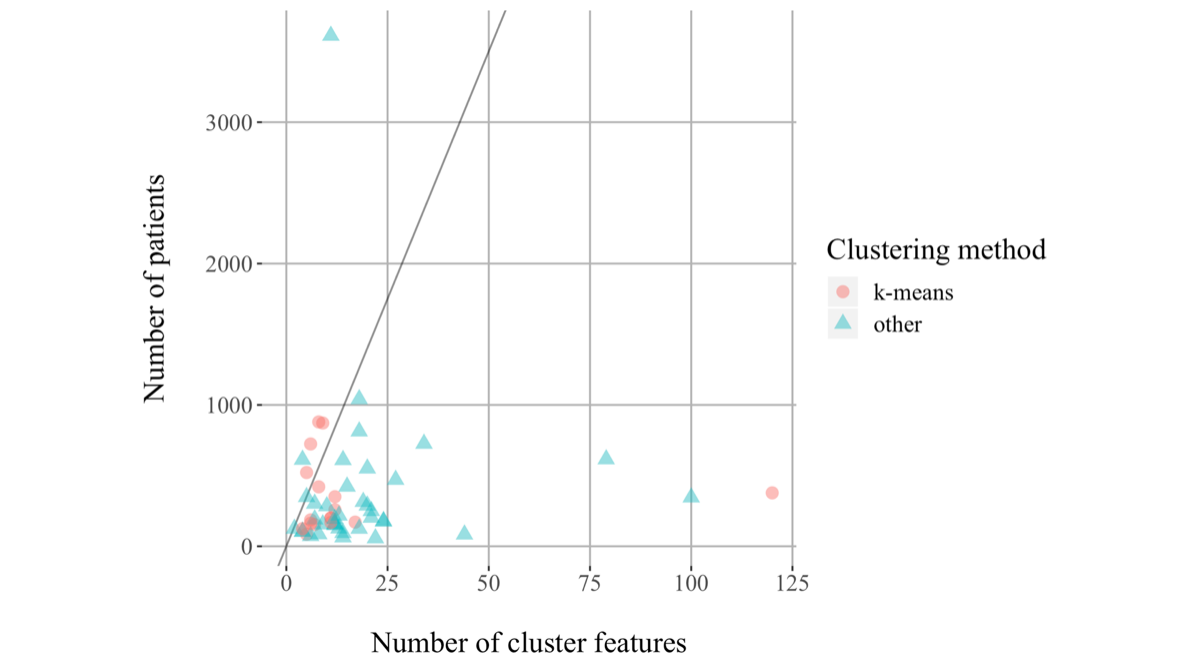
Number of patients versus final number of cluster features. The line corresponds to the number of patients that is equal to 70 times the number of features.

The sample sizes for cluster analysis ranged from 40 to 3612, with a median of 195 patients. Figure 3 presents a scatter plot of the number of patients in the cluster analysis versus the final number of cluster features. The straight line corresponds to the number of samples per feature as recommended by Dolnicar et al [17]. As this estimate was derived from simulation studies using k-means as the clustering method, different markers are used for the studies which used clustering techniques other than k-means. Note that the studies that did not specify the final number of cluster features were omitted from the plot. Six studies (10%) had at least 70 times as many patients as cluster features, as recommended by Dolnicar et al [17].

### Feature Engineering

#### Feature Scaling and Encoding

Judging whether feature scaling and encoding were appropriate depends on the methods of cluster analysis used and vice versa. Therefore, we reported the methods of feature scaling and encoding alongside the methods of cluster analysis in Tables 2-4 and Multimedia Appendix 4.

**Table 2:** Breakdown of methods used by studies applying hierarchical clustering with Ward's linkage (N=23).

Data type, dissimilarity, and scaling of continuous features			Categorical features encoded as binary?	Value, n (%)
**Continuous**				
	**Euclidean assumed**			
		Not detailed	N/A^a^	1 (4)
**Mixed**				
	**Euclidean assumed**			
		Scaled but method unspecified	Yes No	1 (4) 1 (4)
		Scaled to lie in the interval of 0 to 1	Yes	1 (4)
		z-scores	Yes No	1 (4) 1 (4)
		Not detailed	Yes No	3 (13) 6 (26)
	**Euclidean stated**			
		z-scores	Yes No	2 (9) 1 (4)
	**Gower^b^**			
		Gower standardisation	No	3 (13)
		Scaled but method unspecified	No	1 (4)
	**treeClust**			
		Not detailed	No	1 (4)

^a^N/A: not applicable (irrelevant for continuous features).

^b^Computing the Gower coefficient normalizes the distance between feature samples by dividing by the feature range. Therefore, it is not necessary to normalize continuous features prior to computing the Gower coefficient.

**Table 3:** Breakdown of methods used by studies applying k-means (N=22).

Data type, dissimilarity, and scaling of continuous features			Categorical features encoded as binary?	Value, n (%)
**Continuous**				
	**Euclidean assumed**			
		z-scores for one feature	N/A^a^	1 (5)
		No details	N/A	3 (14)
	**Euclidean stated**			
		No details	N/A	1 (5)
**Mixed**				
	**Euclidean assumed**			
		Scaled but method unspecified	No	1 (5)
		z-scores	Yes	6 (27)
		z-scores for one feature	No	1 (5)
		No details	Yes No	1 (5) 2 (9)
	**Euclidean stated**			
		z-scores	Yes	1 (5)
		No details	No	1 (5)
**Unclear**				
	**Euclidean assumed**			
		No details	No	3 (14)
	**Euclidean stated**			
		z-scores	No	1 (5)

^a^N/A: not applicable (irrelevant for continuous features).

**Table 4:** Breakdown of methods used by studies applying SPSS TwoStep (N=7).

Data type, dissimilarity, and scaling of continuous features			Categorical features encoded as binary?	Value, n (%)
**Continuous**				
	**Euclidean assumed**			
		No details	N/A^a^	1 (14)
**Mixed**				
	**Log-likelihood assumed**			
		Scaled to lie in the interval 0 to 1	Yes	1 (14)
		z-scores	No	1 (14)
		No details	Yes	2 (29)
	**Log-likelihood stated**			
		Scaled but method unspecified	No	1 (14)
		No details	No	1 (14)

^a^N/A: not applicable (irrelevant for continuous features).

#### Univariate Feature Transformation

A total 23 (37%) studies applied univariate feature transformation to bring features closer to a normal distribution. The most common univariate feature transformation was logarithmic transformation, applied to nonnormally distributed features in 33% of studies. Lefaudeux et al [30] applied the Box-Cox transformation to all features, whereas Khusial et al [31] stated that data were transformed if necessary but gave no further details.

#### Feature Selection

A total of 22 (35%) studies detailed methods of feature selection to identify their cluster features. The number of features selected in the 63 studies included in this review ranged from 2 to 120, with a median of 12 features. In addition, 47 (75%) studies had mixed-type features, and 12 (19%) had continuous features, and in 4 (6%) studies, the type of features was unclear. Methods for feature selection are listed in Table 5.

A total of 13 (20%) studies used PCA or factor analysis for feature selection. These are not typically methods that should be used for feature selection; we defer further elaboration on the topic for the Discussion. All but one of these studies computed the components (or factors) that represent an underlying latent feature structure, then selected 1 (or in some cases multiple [32,33]) original feature corresponding to each component (or factor) of the latent feature structure. Just et al [34] stated that they used PCA to select features according to statistical significance. As PCA does not involve the computation of statistical significance (*P* values), more detail would be required here to fully understand the methods used for feature selection in this paper. Pérez-Losada et al [35] stated PCA based on Euclidean distances was carried out. It is unclear whether this was an error in reporting or whether PCA was applied to the matrix of Euclidean distances between features instead of the covariance matrix. To implement the latter approach, the Euclidean distances would have to be converted to similarities. Moreover, the authors stated that PCA was used *to identify key clinical components relevant to asthma diagnosis and assessment*. Overall, it is not clear how the authors processed the data using PCA, and there was no justification for using Euclidean distances in that computation. Although the application of PCA leads to the computation of features (principal components) that maximally explain the (remaining) variance in the data, there is no guarantee that the resulting principal components will be highly predictive of an outcome (in this case, asthma diagnosis and assessment).

**Table 5:** Feature engineering methods used in the asthma studies included in this review.

Method		Values, n (%)^a^
**Univariate feature transformation**		
	Logarithmic transformation	21 (33)
	Box-Cox transformation	1 (2)
	Method not explained	1 (2)
**Feature selection**		
	Factor analysis^b^	8 (13)
	Principal component analysis^b^	5 (8)
	Avoid collinearity	3 (5)
	Avoid multicollinearity	3 (5)
	Supervised learning methods	2 (3)
	Multiple correspondence analysis	1 (2)
**Feature transformation**		
	Principal component analysis	4 (6)
	Factor analysis	1 (2)
	Multiple correspondence analysis	1 (2)

^a^As a percentage of all 63 studies.

^b^These are not typically methods of feature selection but have been used in these studies.

Three (5%) studies considered collinearity via pairwise correlations, although the exact criteria for selection features based on this were unclear [36-38]. In addition, 3 (5%) studies avoided multicollinearity, but none detailed their methods for doing so [39-41].

Furthermore, 2 (3%) studies selected features using statistical hypothesis tests with respect to the outcome of interest. Sakagami et al [42] used mean annual decline in forced expiratory volume in 1 second as the outcome feature in a multiple regression analysis using stepwise feature selection. All features with coefficients statistically significantly different to 0 in the multiple regression model were included as cluster features. Seino et al [43] grouped participants according to whether or not they had symptoms of depression. Features were selected for cluster analysis if the difference between the 2 groups (tested using a Wilcoxon rank-sum or chi-square test for continuous and categorical features, respectively) was statistically significant.

#### Feature Transformation

A total of 6 (10%) studies performed feature transformation before cluster analysis; the methods are summarized in Table 5. Of the 4 studies that used PCA for feature transformation, 3 used continuous input features [30,44,45], whereas the fourth used mixed-type input features [46]. None of the studies stated whether the covariance or correlation matrix was used as input for PCA. Only Newby et al [45] specified the number of transformed features retained, and the proportion of original variance accounted for.

Khusial et al [31] performed factor analysis on a subset of the selected features; it is unclear whether categorical features are included in this subset. Although the resulting factors were scaled to z-scores, the authors did not provide further information regarding whether the features were scaled before factor analysis. Four factors were retained, but neither the proportion of variance explained by these factors nor a table of the factor loadings is given.

Sendín-Hernández et al [47] performed MCA to transform 5 continuous and 14 categorical features. They gave the proportion of variance explained by the transformed features but gave neither the number of transformed features retained nor a table of the feature loadings.

### Cluster Analysis

#### Hierarchical Clustering

A total of 23 (37%) studies applied hierarchical clustering with the Ward method [48] as the principal clustering technique. A breakdown of the methods used by these studies is given in Table 2. One study applied these methods to continuous data, and the remaining 22 studies used mixed-type data. Three studies stated that the Euclidean distance was used, 4 used Gower coefficient (issues with the Gower coefficient combined with the Ward method are addressed in the *Discussion* section), and 1 used tree-based dissimilarity measure [49]. For the remaining 15 studies, we assumed that the Euclidean distance was used. Of the 23 studies, 11 did not detail whether the features were rescaled. Of the 17 studies using the Euclidean distance with mixed-type features, 8 encoded categorical features as binary features.

A total of 3 (5%) further studies (in addition to the 23 studies introduced at the start of the paragraph) applied hierarchical clustering to continuous data. Amore et al [39] used the average linkage and the Euclidean distance, whereas 2 studies used hierarchical clustering but did not specify the linkage or dissimilarity measure used [44,50].

#### k-Means

A total of 22 (35%) studies used k-means clustering as the principal clustering technique. A breakdown of the methods used by these 3 studies is given in Multimedia Appendix 4. A breakdown of the methods used by these studies is given in Table 3. Five studies applied k-means to continuous data, and 13 studies applied it to mixed-type data. In 3 studies, the cluster features were not explicitly stated, and the data types therefore were unclear. Of the 22 studies, 4 explicitly stated that the Euclidean distance was used. As no other dissimilarity metrics were mentioned, we assumed that the Euclidean distance was used in the remaining 18 studies because it is often the default option for most algorithmic packages. Of the 22, 11 studies did not detail whether continuous features were scaled before cluster analysis. Of the 13 studies with mixed-type data, 8 encoded categorical features as binary features.

#### Preclustering Methods

When dealing with very large sample sizes, it can be advantageous to introduce a precluster step. The aim is to group samples and to use these groups or *preclusters* as input to a follow-on clustering algorithm (ie, using 2 steps with cascaded cluster algorithms). This step is used to reduce the computation time required to compute the cluster results.

A total of 7 (11%) studies used the SPSS TwoStep clustering method [51,52]. A breakdown of the preprocessing methods and distance measures used by these studies is given in Table 4. In the first (precluster) step, a cluster feature tree is identified. In the second step, the preclusters are merged stepwise until all clusters are in 1 cluster using the Euclidean or log-likelihood distance for continuous or mixed-type features, respectively. An advantage of the log-likelihood distance measure is that it is designed to handle mixed-type features. However, in doing so, it assumes that continuous (categorical) features follow a normal (multinomial) distribution within clusters.

None of the studies in this review adequately considered the distributional assumptions made by the SPSS TwoStep method. Ruggieri et al [53] acknowledged that the method assumes continuous features are normally distributed, but they did not explicitly report whether these assumptions were satisfied. Although Newby et al [45] acknowledged that the method assumes cluster features are statistically independent within clusters, they only go as far as to ensure that their cluster features are uncorrelated (by applying PCA), which does not necessarily imply independence. The remaining 5 studies that used the SPSS TwoStep method did not reference distributional assumptions.

Two (3%) further studies preclustered samples (Just et al [34] specified k-means, and Ye et al [54] did not specify the precluster method) and then applied hierarchical clustering with the Ward linkage method on the preclusters. A breakdown of the methods used by these 2 studies is given in Multimedia Appendix 4.

#### k-Medoid Methods

Three studies used k-medoid methods. A breakdown of the methods used by these 3 studies is given in Multimedia Appendix 4. Two used k-medoids implemented by the Partition Around Medoids algorithm [55]. Lefaudeux et al [30] used the Euclidean distance with center-scaled continuous data, and Sekiya et al [56] used the Gower metric with mixed-type data. Loza et al [57] applied fuzzy partition-around-medoid clustering with the Euclidean distance to continuous data scaled with average absolute deviation.

#### Kernel k-Means and Spectral Clustering

Kernel k-means and spectral clustering are different but related methods, which may be used to identify clusters that are not linearly separable in the input feature space [58]. As these methods were used by only 1 study each (Wu et al used multiple kernel k-means [59], and Howrylak et al used spectral clustering [37]), we do not explore them in detail in this review. However, details of the feature scaling, encoding, and distance measures used by these 2 studies is given in Multimedia Appendix 4.

#### Unclear Methods

Wang et al [41] described a 2-step clustering method in which the first step was to carry out hierarchical clustering using the Ward method, but with the log-likelihood distance in place of the Euclidean distance. This first step was used to determine the number of clusters, which was then used in the k-means method in the second step. However, the authors cite the SPSS TwoStep method [52], which is different from that described previously. It was therefore ambiguous which clustering method was applied in this study.

### Postprocessing

#### Choosing the Number of Clusters

A total of 54 (86%) studies explained in detail the methods used to select the number of clusters. Of these, 20 (32%) studies used more than one method for choosing the number of clusters. The maximum number of methods used was 6.

A total of 27 (43%) studies used a dendrogram to choose the number of clusters to include in their study (Table 6). Note that 18 of the 22 studies that applied k-means clustering used hierarchical cluster as a first step to identify the likely number of clusters. Of these 18 studies, 11 explicitly stated that the dendrogram was used to choose the number of clusters.

Of the 8 (13%) studies that specified a maximum number of clusters, the maximum number ranged between 2 and 15 clusters. Seven (11%) studies used a statistic (or multiple statistics), including the c-index [60], Gap statistic [37], deviation from ideal stability [30], Calinski and Harabasz index [30], Dunn’s partition [57], cubic cluster criterion (CCC) statistic [28], pseudo-F statistic [28,36], and pseudo-T2 statistic [28,36].

Four studies (6%) avoided very small clusters. Approaches to this include merging 2 clusters containing 6 and 12 samples [61], omitting small clusters containing 1 [35] and 6 [62] samples, and choosing the number such that no cluster contained less than 10% of the total samples [63].

#### Stability

A total of 11 (17%) studies tested the stability of their cluster solution; the methods are detailed in Table 6. Of these, 1 study used 2 methods, and the remaining 10 each used only 1 method to test stability.

#### Quality

A total of 24 (38%) studies assessed the quality of their solution using methods beyond those assessing stability. The methods are detailed in Table 6. Of these, 3 used more than one method. The maximum number of methods used in this study was 4.

Of the 30 studies that assessed the stability or quality of their cluster analysis, 21 (70%) reported their findings. However, the reporting of these results was in many cases brief, consisting of statements such as “the clusters were shown to be stable” without providing supporting evidence.

**Table 6:** Postprocessing methods used in the asthma studies included in this review.

Method		Values, n (%)^a^
**Choosing the number of clusters**		
	Dendrogram	27 (43)
	Hierarchical clustering with Ward linkage	19 (30)
	Specify a maximum number of clusters^b^	8 (13)
	Statistic(s)	7 (11)
	Silhouette plot or average silhouette width	5 (8)
	Bayesian information criterion	4 (6)
	Specify a minimum size of smallest cluster^b^	4 (6)
	Previous studies	3 (5)
	Unclear	3 (5)
	Clinical interpretation	2 (3)
	Scree plot	1 (2)
**Stability**		
	Repeated in random subset	3 (5)
	Leave-one-out cross-validation	3 (5)
	Bootstrap methods	3 (5)
	Unclear methods	2 (3)
	Train and test set	1 (2)
**Quality**		
	Repeated in selected subset	8 (13)
	Repeated with difference methods	6 (10)
	Repeated with different initial configurations	5 (8)
	Repeated in separate cohort	4 (6)
	Repeated with altered features	3 (5)
	Repeated at different time point	3 (5)
	Repeated with different software	1 (2)

^a^Studies may have used more than 1 method.

^b^These methods were not included when calculating the number of methods used to choose the number of clusters.

## Discussion

### Principal Findings

We identified 63 studies that applied cluster analysis to multimodal clinical data to identify subtypes of asthma. We explored the clustering methodologies and their limitations in detail. The principal finding of this review was that the majority of the reviewed studies have flaws in the application of cluster analysis. Although some of these flaws were related to the multimodal nature of the clinical data, they extended to aspects of cluster analysis, which are agnostic of data type, such as sample size, stability, and reporting of the results.

These findings build on a previous review, which identified limitations such as lack of robustness in feature selection and neglect to specify distance measures in studies using cluster analysis to contribute to our understanding of the spectrum of asthma syndrome [11]. Our review investigated the methods of feature engineering more generally and identified not only neglect to specify dissimilarity measures but also instances in which the dissimilarity measure was inappropriate for the data to which it was applied. In addition, we identified issues related to sample size, cluster analysis methods, choosing the number of clusters, and testing the stability and quality of results. These issues are discussed in the following paragraphs.

A widespread limitation in the reviewed studies was the small sample size. Studies had overall sample sizes as small as 40 patients, with clusters as small as 6 patients. We argue that there is limited utility in clustering data with such small sample sizes: they may result in clusters that are unstable [64] and may therefore lead to selecting fewer clusters than are present in the underlying population from which the data are sampled.

In the following paragraphs, we discussed the limitations of 3 of the feature selection approaches applied by the reviewed studies. The first approach was to avoid collinearity or multicollinearity or excluding features that were considered to be *clinically redundant*. Although one should avoid including features that are *redundant* (can be completely deduced from a combination of the other cluster features), this is rarely the case. Therefore, removing features inevitably leads to loss of information. We suggest that the removal of features based on redundancy needs to be carefully considered, for example, 2 or more features (some of which may appear univariately redundant) may jointly contribute toward determining a cluster (or similarly toward the estimation of a clinical outcome in a standard supervised learning setup).

The second was the use of PCA or factor analysis to select features, which has a similar motivation to the concept described earlier for discarding statistically correlated features. There are methodological justifications for the use of PCA, factor analysis, or other nonlinear embedding methods for feature transformation [19]. They aim to jointly combine the original features and project them in a new feature space, which may have some useful properties, including interpretation, determining latent feature structure, and improving the clustering or statistical mapping outcomes [16]. However, we suggest exercising caution toward using these methods for feature selection as described in some of the studies summarized in the Results section of this review because they were fundamentally developed toward different aims. Haldar et al used PCA for feature selection in the first publication to apply cluster analysis to identify asthma subtypes [14]. It is possible that other studies used this as a point of reference for these methods, leading to the common application of these methods in the field of asthma subtyping.

The third approach to feature selection was the use of statistical hypothesis tests with respect to outcomes of interest, as done in 2 studies [42,43]. Methods in which an outcome of interest is used to guide feature selection in cluster analysis have been described previously [65,66]. Although these approaches may be useful for situations in which there exists an outcome of particular interest to the clustering problem, the user should be aware of and acknowledge the assumptions made in the process. In the context of the 2 reviewed studies that used this approach, Sakagami et al did not acknowledge the linearity assumption in linear regression [42], whereas Seino et al’s method does not account for potentially highly correlated features [43], a concept that is key in feature selection for cluster analysis.

Feature transformation was applied in only 6 studies, and the methods were generally poorly reported. As with cluster analysis, feature encoding and scaling are important considerations in feature transformation, but none of the studies gave adequate details in their methods. The results of feature transformation were also poorly reported. Although the key reason for applying feature transformation methods is to reduce the dimensionality of the dataset, only 2 [31,45] of the 6 studies provided details on the number of features retained. We suggest that the results of PCA, factor analysis, or MCA should include a table of component (or factor) loadings, the number of features retained, and the proportion of variance accounted for in the transformed features.

Most studies explicitly stated the clustering method that they used but were less explicit regarding the preprocessing steps and choice of dissimilarity measure. Hastie et al [16] state, “Specifying an appropriate dissimilarity measure is far more important in obtaining success with clustering than choice of clustering algorithm.”

We expand on this statement, further adding that preprocessing steps such as feature scaling and feature encoding are also more important in obtaining success than the choice clustering algorithm. This is in line with the conclusions of Prosperi et al, who demonstrated that clustering using different feature sets and encodings in asthma datasets can lead to different cluster solutions [29]. Both preprocessing steps and dissimilarity measures, along with their relation to clustering algorithms, have been given poor consideration in clustering applications in asthma, as discussed in the following 3 paragraphs.

First, the Euclidean distance was used with mixed-type data in over half of the studies (54%). Although the Euclidean distance is intended for use with continuous data, problems associated with applying it to mixed-type data may be mitigated by carefully considering feature scaling and feature encoding. However, in our review, we found that many studies did not specify their methods for rescaling, and many studies included ordinal and nominal categorical features but did not specify how these would be treated when calculating the Euclidean distances. The lack of consideration of feature scaling and encoding in these cases may have resulted in assigning an unintended weight structure to the cluster features.

Second, 4 studies used Gower coefficient in hierarchical clustering with Ward linkage [36,67-69], and 1 used tree-based distances [49,70]. These studies should be given some credit for using dissimilarities that can handle mixed-type data. However, the application of hierarchical clustering with Ward linkage relies on the properties of the Euclidean distance in the computations. These properties do not hold for Gower coefficient, and hence, errors are perpetuated at each level of the hierarchy. An example that demonstrates this issue is given in Multimedia Appendix 5.

A final point in the use of k-means and hierarchical clustering using the Ward method with mixed cluster features is that the theory underpinning these methods involves the calculation of cluster means. The mean is not an appropriate summary statistic for categorical features, which are more typically summarized by the mode. For this reason, we suggest that k-medoids may be a more appropriate method for mixed-type features used in clustering. Instead of computing each cluster’s mean (as with hierarchical clustering using Ward’s method and k-means), k-medoids compute each cluster’s medoid, defined as the sample in the cluster for which the average dissimilarity to all other samples in the cluster is minimized [55]. In addition, k-medoids do not rely on the properties of the Euclidean distance in the computations, thus avoiding the issue described in the previous paragraph. Despite these advantages, only 2 studies in this review used k-medoids [30,56].

The SPSS TwoStep method was used in 7 of the 63 studies investigated here. We see 2 key limitations with the application of this method across the reviewed studies. First, none of the studies gave adequate consideration to the distributional assumptions made when using the log-likelihood distance, and most did not mention the assumptions at all. Second, this method is designed for clustering several millions of samples with many features within an acceptable time and makes a key compromise in doing so [52]. This compromise is that the data are not stored in the main memory but are read sequentially, hence making the solution sensitive to the ordering of the data. None of the studies acknowledged this inherent shortcoming, nor did they confirm that their data were in a random order. Perhaps, more concerningly, the studies that applied these methods actually had very small datasets (range 84-349 samples) that could easily be stored, therefore making other standard techniques more appropriate. In our view, this compromise was therefore unnecessary.

Only 1 study [57] used a method that obtains a *fuzzy* cluster solution (in which a patient may be assigned a membership value to multiple clusters), as opposed to a *hard* cluster solution (in which each patient is assigned to a single cluster) [23]. A fuzzy cluster solution can indicate where a patient membership value is similar across multiple clusters, whereas this information is lost (or leads to lack of stability) in a hard cluster solution. Owing to the noisy nature of clinical data and the clinical complexity of grouping patients into distinct groups, we suggest that fuzzy cluster solutions may be more appropriate than hard cluster solutions in the review applications in asthma. However, it is important to acknowledge that there are added challenges in the interpretation and communication of fuzzy cluster solutions and that the methods may be more computationally intensive [71].

Selecting the number of clusters can be challenging and depends largely on the context of the application. In the case of the reviewed applications in asthma, the *true* number of clusters is unknown, and the analyses are exploratory. Although 86% of the review studies gave some details regarding their methods for choosing the number of clusters (*k*), they were generally poorly reported. The most popular approach was the dendrogram, but only Labor et al [72] specified their criteria for cutting the dendrogram. In 14 studies, the dendrogram was the only method mentioned. We suggest that more than one method should be used to select the number of clusters to validate this decision.

Our review shows that studies rarely tested the stability and quality of their results, with a particular lack of emphasis on stability. This is concerning, as many studies use methods such as k-means, which reach local minima, and apply them to small sample sizes, thus increasing the risk of obtaining unstable results. We argue that because of the unsupervised nature of cluster analysis, testing the stability and quality of the results should be a key theme and would like to urge researchers and peer reviewers in this research field to carefully consider these aspects. However, we do appreciate that assessing the stability and quality of a solution in the absence of *ground truth* is challenging and that there are currently no well-established frameworks for doing so [27].

Although this review focused on applications in subtyping asthma, the identified issues have been found in studies using cluster analysis to subtype other diseases. For example, recent studies in autism [73] and hypersomnolence [74] have applied cluster analysis to very small samples (55 and 17 patients, respectively). A recent study on Parkinson disease [75] stated in the main text that a *model-based* cluster analysis method was used, whereas the supplementary materials revealed that the method was in fact k-means, which is not model-based. In addition, supplementary materials listed 3 methods for choosing the number of clusters (CCC, pseudo-F, and R-squared statistics) but did not present the results from these 3 methods anywhere in the main text or supplementary materials. These findings demonstrate the widespread nature of the issues that this review has highlighted, and that the issues are not restricted to asthma-related studies.

For a recent example of a well-considered and well-reported application of cluster analysis to multimodal clinical data, we refer the reader to Pikoula et al’s study of Chronic Obstructive Pulmonary Disease subtypes [76]. The main text and supplementary materials provide a transparent report of the methodology with respect to feature engineering and cluster analysis methods. In particular, Pikoula et al performed a rigorous assessment of the stability, reproducibility, and sensitivity of the resulting clusters, which could be used as a framework for future studies. The results that were key to the study’s conclusions (eg, MCA feature loadings, silhouette plots, results from stability, reproducibility, and sensitivity analyses) are correctly reported in the manuscript, enabling readers to have a thorough understanding of the study’s findings.

### Limitations

The literature search presented in this study is comprehensive but practically cannot be exhaustive. We restricted the search to articles that included the terms *cluster analysis* or clustering*. Although it is not strictly speaking correct to do so, some studies in the medical literature use the term *classification* to refer to cluster analysis, often confusing the 2 terms and sometimes using them almost interchangeably, for example, see the studies by Just et al [34] and Kim et al [46]. Widening the search to identify studies that use the term *classification* would have greatly increased the initial number of results of the PubMed search, but we suspect that the increase in the number of eligible studies for cluster analysis identified would have been small. Similarly, the terms *latent class analysis* and *mixture model analysis* might sometimes be erroneously used to refer to cluster analysis: we clarify that these terms were not included in our search strategy. As this is not a systematic review, we feel that our search criteria are fully sufficient for this study’s purposes.

We did not fully explore multiple kernel k-means [77] or spectral clustering [78] methods, each used by 1 study in this review. As with all other cluster analysis methods mentioned here, careful consideration must be taken when applying these methods to mixed-type data. There are numerous other considerations that are important to these methods, such as the choice of kernel function, but these are beyond the scope of this review.

### Conclusions

This review highlights a number of issues in previous applications of cluster analysis to multimodal clinical data in asthma. We make the following key recommendations based on these findings:

- Careful consideration should be given to the preprocessing of multimodal clinical data and how the scaling and encoding of features may affect their weighting in the analysis.
- The choice of dissimilarity measures and cluster analysis methods are dependent on one another as well as on the scaling and encoding of the data. Certain combinations of these data analytics components may be incompatible and give unreliable results.
- The stability and quality of the cluster results should be thoroughly evaluated.

The abovementioned recommendations focus on the application of cluster analysis, but we put similar emphasis on the clear reporting of each of the abovementioned points, as this was also found to be lacking in the reviewed papers.

## Appendix

Multimedia Appendix 1 Preferred Reporting Items for Systematic Reviews and Meta-Analyses checklist.

Multimedia Appendix 2 Data dictionary.

Multimedia Appendix 3 Study characteristics.

Multimedia Appendix 4 Breakdown of methods used by the 11 studies that did not use the three most common clustering methods.

Multimedia Appendix 5 Illustrative example of the use of Gower coefficient with hierarchical clustering and Ward linkage.

## Abbreviations

CCC: cubic cluster criterion
HDR: Health Data Research
MCA: multiple correspondence analysis
PCA: principal component analysis
PRISMA: Preferred Reporting Items for Systematic Reviews and Meta-Analyses
