# Supplementary material for: Challenges of Clustering Multimodal Clinical Data: Review of Applications in Asthma Subtyping
Source: JMIR Med Inform. 2020 May 28;8(5):e16452. doi: 10.2196/16452 (PMC7290450; doi:10.2196/16452)
Supplement: Multimedia Appendix 2 [file medinform_v8i5e16452_app2.docx]

Multimedia Appendix 2

# Data Dictionary

The following table contains the names of all items for which data were extracted, along with a short description. Items are grouped according to the step of cluster analysis.

Some items take values yes/no, e.g. ‘Was the study identified in the PubMed search?’

- yes = it was identified
- no = it was not identified

Some items take values yes/no/NA, e.g. ‘Were smokers exluded?’

- yes = the article states that smokers were excluded
- no = the article states that smokers were not excluded
- NA = the article does not mention whether smokers were excluded

Some items take the values yes/NA, e.g. ‘Complete case analysis’

- yes = the article states that a complete case analysis was carried out
- NA = the article does not mention a complete case analysis (but we cannot categorically say that it was not as this may just be poor reporting)

| Item group | Item name | Description of item |
| --- | --- | --- |
| General study information | author | First author on study |
|  | year | Year of publication |
|  | journal | Publication journal |
|  | id | Unique study ID |
|  | pubmed | Was the study identified in the PubMed search? yes/no |
|  | scopus | Was the study identified in the Scopus search? yes/no |
|  | date_extract | Date of extraction |
|  | population | Study population used in the cluster analysis |
|  | country | Country of origin of study population |
| Details regarding exclusion criteria | exc_smoke | Were smokers were excluded? yes/no/NA |
|  | exc_copd | Were patients with Chronic Obstructive Pulmonary Disease (COPD) were excluded? yes/no/NA |
| Methods for chosing candidate cluster features | clinical_easy | Were candidate cluster features chosen on the basis of being easy to measure in clinical practice? yes/NA |
|  | clinical_previous | Were candidate cluster features chosen on the basis of previous studies? yes/NA |
|  | clinical_redundance | Were candidate cluster features chosen on the basis of avoiding clinical redundancy? yes/NA |
|  | clinical_relevence | Were candidate cluster features chosen on the basis of their relevance to asthma subtypes? yes/NA |
| Method for dealing with missing data | miss_cc | Complete case analysis: yes/NA |
|  | miss_handle | Cluster analysis methods could handle missing data: yes/NA |
|  | miss_impute | Imputation: yes/NA |
|  | miss_none | No missing data present: yes/NA |
|  | miss_pat | Patients with >x% missing data removed: yes/NA |
|  | miss_var | Features with >x% missing values removed: yes/NA |
| Patients included in cluster analysis | n_pat | Number of patients |
| Univariate variable transformations | ut_box | Box-cox power transformation: yes/NA |
|  | ut_log | Log-transformation: yes/NA |
|  | ut_unclear | Univariate feature transformation carried out but methods unclear: yes/NA |
| Method of feature selection | vselect_coll | Avoid collinearity: yes/NA |
|  | vselect_fa | Factor analysis: yes/NA |
|  | vselect_mca | Multiple correspondence analysis: yes/NA |
|  | vselect_multico | Avoid multicollinearity: yes/NA |
|  | vselect_pca | Principal component analysis: yes/NA |
|  | vselect_supervised | On the basis of statistical tests: yes/NA |
| Methods of feature transformation | ft_factor | Factor analysis: yes/NA |
|  | ft_mca | Multiple correspondence analysis: yes/NA |
|  | ft_pca | Principal component analysis: yes/NA |
| Cluster feature details | data_type | Type of cluster features: categorical/continuous/mixed/unclear |
|  | n_cat_feat | Number of categorical cluster features |
|  | n_cont_feat | Number of continuous cluster features |
|  | n_feat | Total number of cluster features |
|  | encode_bin | Were all categorical variables encoded as binary variables: yes/NA |
| Methods used to scale the features | scale_01 | Scaled to the range [0,1]: yes/NA |
|  | scale_aad | Average absolute deviation: yes/NA |
|  | scale_centre | Centre-scaled: yes/NA |
|  | scale_gower | Gower standardisation: yes/NA |
|  | scale_unit | Scaled to unit length: yes/NA |
|  | scale_unspec | Standardised but method unspecified: yes/NA |
|  | scale_z | z-scores: yes/NA |
|  | scale_z_one | One feature scaled to z-scores: yes/NA |
| Dissimilarity measure used | prox_euc_ass | Euclidean assumed due to cluster analysis method: yes/NA |
|  | prox_euc_ex | Euclidean stated: yes/NA |
|  | prox_gower | Gower’s coefficient: yes/NA |
|  | prox_ll_ass | Log-likelihood measure assumed due to cluster analysis method: yes/NA |
|  | prox_ll_exp | Log-likelihood measure stated: yes/NA |
|  | prox_spearman | Dissimilarity measure based on Sprearman’s Rho: yes/NA |
|  | prox_tree | Tree-based method using the treeClust R package: yes/NA |
| Methods of cluster analysis | cluster_fuzzypam | Fuzzy Partition Around Medoids: yes/NA |
|  | cluster_hier | Hierarchical clustering with no details of linkage: yes/NA |
|  | cluster_hier_ave | Hierarchical clustering with average linkage: yes/NA |
|  | cluster_kmean | k-means: yes/NA |
|  | cluster_kmean_ward | k-means for pre-clusters, hierarchical with Ward’s linkage for final clusters: yes/NA |
|  | cluster_kmedoid | k-medoids: yes/NA |
|  | cluster_mmlmkkc | Multiple kernel k-means: yes/NA |
|  | cluster_pre_hier | Pre-clustering followed by hierarchical clustering: yes/NA |
|  | cluster_spec | Spectral clustering: yes/NA |
|  | cluster_spss | SPSS TwoStep: yes/NA |
|  | cluster_unclear | Unclear clustering methods: yes/NA |
|  | cluster_ward | Hierarchical clustering with Ward’s linkage: yes/NA |
| Method for choosing the number of clusters | k_bic | Bayesian Information Criterion (Implemented as part of SPSS Two Step): yes/NA |
|  | k_dend | Dendrogram (explicitly stated or diagram included, stating hierarchical techniques not sufficient): yes/NA |
|  | k_interp | Clinical interpretation of the cluster solution: yes/NA |
|  | k_max | Did the study specify the maximum number of clusters which they considered?: yes/NA |
|  | details_k_max | What was the maximum number of clusters considered? |
|  | k_mpc | Did the study state the minumum size of cluster which they accept? yes/NA |
|  | details_k_mpc | What was the minimum size of cluster considered? |
|  | k_previous | Previous studies/results: yes/NA |
|  | k_scree | Scree plot: yes/NA |
|  | k_silhouette | Silhouette plot: yes/NA |
|  | k_stat | Statistic(s): yes/NA |
|  | details_k_stat | Which statistic(s) were used? |
|  | details_k_stat_n | How many statistics were used? |
|  | k_unclear | Unclear methods for choosing the number of clusters: : yes/NA |
|  | k_wards | Hierarchical clustering with Ward’s linkage: yes/NA |
|  | n_k | Number of clusters chosen |
| Method for testing quality of clusters | rep_initial | Repeat with different initial values: yes/NA |
|  | rep_method | Repeat with different methods: yes/NA |
|  | rep_select | Repeat in a selected subset: yes/NA |
|  | rep_sep | Repeat in a separate cohort: yes/NA |
|  | rep_software | Repeat with different software: yes/NA |
|  | rep_time | Repeat at different time-point: yes/NA |
|  | rep_var | Repeat with altered variables: yes/NA |
| Method for testing the stability of the clusters | stab_boot | Bootstrap methods: yes/NA |
|  | stab_loo | Leave-one-out cross-validation: yes/NA |
|  | stab_random | Repeat in random subset: yes/NA |
|  | stab_tt | Split into train and test set: yes/NA |
|  | stab_unclear | Methods unclear: yes/NA |
| Reporting | details_report_stab | Were the results of testing the stability/quality of the solution reported: yes/no/NA |
