# Supplementary material for: Challenges of Clustering Multimodal Clinical Data: Review of Applications in Asthma Subtyping
Source: JMIR Med Inform. 2020 May 28;8(5):e16452. doi: 10.2196/16452 (PMC7290450; doi:10.2196/16452)
Supplement: Multimedia Appendix 3 [file medinform_v8i5e16452_app3.docx]

Multimedia Appendix 3

Characteristics of the 63 studies included in the review with references.

| **First author** | **Year** | **Study population** | **N^a^** | **M^b^** | **Reference** |
| --- | --- | --- | --- | --- | --- |
|  |  |  |  |  |  |
| Agache | 2010 | Adults with asthma and seasonal allergic rhinitis | 57 | NA | [1] |
| Agache | 2016 | Adults with asthma | 64 | 14 | [2] |
| Amelink | 2013 | Adult-onset asthma | 200 | 11 | [3] |
| Amore | 2013 | Adults with asthma | 104 | 4 | [4] |
| Baptist | 2018 | Older adults with asthma | 180 | 24 | [5] |
| Benton | 2010 | Children with asthma | 154 | 7 | [6] |
| Bourdin | 2014 | Adults with severe asthma | 112 | NA | [7] |
| Cabral | 2017 | Children with asthma | 289 | 20 | [8] |
| Deliu | 2018 | Children with asthma | 613 | 4 | [9] |
| Ding | 2018 | Children with asthma | 813 | 18 | [10] |
| Fitzpatrick | 2011 | Children with difficult asthma | 161 | 12 | [11] |
| Gomez | 2017 | Adults with asthma | 156 | 12 | [12] |
| Gupta | 2010 | Adults with severe asthma | 99 | NA | [13] |
| Haldar | 2008 | Adults with refractory asthma | 187 | 6 | [14] |
| Howrylak | 2014 | Children with mild-moderate asthma | 1041 | 18 | [15] |
| Hsiao | 2019 | Adults with asthma | 421 | 8 | [16] |
| Ilmarinen | 2017 | Adult-onset asthma | 171 | 17 | [17] |
| Jang | 2013 | Adults with refractory asthma | 86 | 5 | [18] |
| Just | 2012 | Children with persistent asthma | 315 | 19 | [19] |
| Just | 2012 | Children with asthma | 551 | 20 | [20] |
| Just | 2013 | Children with asthma | 150 | 12 | [21] |
| Just | 2014 | Children with allergic asthma | 125 | 18 | [22] |
| Kaneko | 2013 | Adults with asthma | 880 | 8 | [23] |
| Khusial | 2017 | Adults with asthma | 611 | 14 | [24] |
| Kim | 2013 | Adults with asthma | 724 | 6 | [25] |
| Kim | 2017 | Adults with asthma | 259 | 12 | [26] |
| Konno | 2018 | Adults with severe asthma | 127 | 13 | [27] |
| Konstantellou | 2015 | Adults with asthma | 170 | NA | [28] |
| Labor | 2018 | Adults with asthma | 201 | 11 | [29] |
| Lavoie-Charland | 2013 | Adults with asthma | 522 | 5 | [30] |
| Lee | 2017 | Adults with nerd | 302 | 7 | [31] |
| Lefaudeux | 2017 | Adults with asthma | 266 | NA | [32] |
| Lemiere | 2014 | Adults with occupational asthma | 73 | 6 | [33] |
| Loureiro | 2015 | Adults with asthma | 57 | 22 | [34] |
| Loza | 2016 | Adults with asthma | 156 | 9 | [35] |
| Mahut | 2011 | Children with asthma | 169 | 11 | [36] |
| Meyer | 2014 | Adults with asthma | 191 | 7 | [37] |
| Meyer | 2014 | Adults with asthma | 195 | 12 | [38] |
| Moore | 2010 | Adults with asthma | 726 | 34 | [39] |
| Moore | 2014 | Adults with asthma | 423 | 15 | [40] |
| Newby | 2014 | Adults with refractory asthma | 349 | 5 | [41] |
| Park | 2015 | Elderly adults with asthma | 872 | 9 | [42] |
| Patrawalla | 2012 | Adults with asthma | 471 | 27 | [43] |
| Pérez-Losada | 2018 | Children with asthma | 163 | 6 | [44] |
| Qiu | 2018 | Adults with acute exacerbation | 218 | 13 | [45] |
| Ruggierei | 2017 | Children with asthma | 125 | 2 | [46] |
| Sakagami | 2014 | Adults with asthma | 86 | 8 | [47] |
| Schatz | 2014 | Adults with asthma | 3612 | 11 | [48] |
| Seino | 2018 | Adults with asthma and depressive symptoms | 95 | 14 | [49] |
| Sekiya | 2016 | Adults with severe or life-threatening exacerbation | 175 | 24 | [50] |
| Sendín-Hernández | 2018 | Adults and adolescents with allergic asthma | 225 | NA | [51] |
| Serrano-Pariente | 2015 | Adults with life-threatening exacerbation | 84 | 44 | [52] |
| Su | 2018 | Children with asthma | 351 | 12 | [53] |
| Sutherland | 2012 | Adults with persistent asthma | 250 | 21 | [54] |
| Tsukioka | 2017 | Athletes with asthma | 104 | 4 | [55] |
| Ueno | 2019 | Adults with recent exacerbation | 100 | NA | [56] |
| Wang | 2017 | Adults with asthma | 284 | 10 | [57] |
| Wu | 2014 | Adults with asthma | 378 | 120 | [58] |
| Wu | 2019 | Adults with asthma | 346 | 100 | [59] |
| Ye | 2017 | Adults and adolescents with moderate-to-severe asthma | 203 | 21 | [60] |
| Youroukova | 2017 | Adults with moderate-to-severe asthma | 40 | NA | [61] |
| Zaihra | 2016 | Adults with moderate-to-severe asthma | 125 | 4 | [62] |
| Zoratti | 2016 | Children with asthma | 616 | 79 | [63] |

^a^ N refers to the number of samples
^b^ M refers to the number of cluster feature

# References

1. Agache I, Ciobanu C. Risk factors and asthma phenotypes in children and adults with seasonal allergic rhinitis. Phys Sportsmed 2010;38(4):81–86. [doi: 10.3810/psm.2010.12.1829]

2. Agache I, Strasser DS, Klenk A, Agache C, Farine H, Ciobanu C, Groenen PMA, Akdis CA. Serum IL-5 and IL-13 consistently serve as the best predictors for the blood eosinophilia phenotype in adult asthmatics. Allergy Eur J Allergy Clin Immunol 2016;71(8):1192–1202. PMID:27060452

3. Amelink M, De Nijs SB, De Groot JC, Van Tilburg PMB, Van Spiegel PI, Krouwels FH, Lutter R, Zwinderman AH, Weersink EJM, Ten Brinke A, Sterk PJ, Bel EH. Three phenotypes of adult-onset asthma. Allergy Eur J Allergy Clin Immunol 2013;68(5):674–680. PMID:23590217

4. Amore M, Antonucci C, Bettini E, Boracchia L, Innamorati M, Montali A, Parisoli C, Pisi R, Ramponi S, Chetta A. Disease Control in Patients with Asthma is Associated with Alexithymia but not with Depression or Anxiety. Behav Med 2013;39(4):138–145. [doi: 10.1080/08964289.2013.818931]

5. Baptist AP, Hao W, Karamched KR, Kaur B, Carpenter L, Song PXK. Distinct Asthma Phenotypes Among Older Adults with Asthma. J Allergy Clin Immunol Pract [Internet] Elsevier Inc; 2018;6(1):244-249.e2. [doi: 10.1016/j.jaip.2017.06.010]

6. Benton AS, Wang Z, Lerner J, Foerster M, Teach SJ, Freishtat RJ. Overcoming heterogeneity in pediatric asthma: Tobacco smoke and asthma characteristics within phenotypic clusters in an African American cohort. J Asthma 2010;47(7):728–734. PMID:20684733

7. Bourdin A, Molinari N, Vachier I, Varrin M, Marin G, Gamez AS, Paganin F, Chanez P. Prognostic value of cluster analysis of severe asthma phenotypes. J Allergy Clin Immunol 2014;134(5):1043–1050. PMID:24985405

8. Cabral ALB, Sousa AW, Mendes FAR, Carvalho CRF de. Phenotypes of asthma in low-income children and adolescents: cluster analysis. J Bras Pneumol [Internet] 2017;43(1):44–50. PMID:28125150

9. Deliu M, Yavuz TS, Sperrin M, Belgrave D, Sahiner UM, Sackesen C, Kalayci O, Custovic A. Features of asthma which provide meaningful insights for understanding the disease heterogeneity. Clin Exp Allergy Wiley/Blackwell (10.1111); 2018 Sep 15;48:1. [doi: 10.1111/cea.13014]

10. Ding L, Li D, Wathen M, Altaye M, Mersha TB. African ancestry is associated with cluster- based childhood asthma subphenotypes. BMC Medical Genomics; 2018;1–11. [doi: 10.1186/s12920-018-0367-5]

11. Fitzpatrick AM, Teague WG, Meyers DA, Peters SP, Li X, Li H, Wenzel SE, Aujla S, Castro M, Bacharier LB, Gaston BM, Bleecker ER, Moore WC. Heterogeneity of severe asthma in childhood: Confirmation by cluster analysis of children in the National Institutes of Health/National Heart, Lung, and Blood Institute Severe Asthma Research Program. J Allergy Clin Immunol [Internet] Elsevier Ltd; 2011;127(2):382-389.e13. PMID:21195471

12. Gomez JL, Yan X, Holm C, Grant N, Liu Q, Cohn L, Nezgovorova V, Meyers DA, Bleecker ER, Crisafi GM, Jarjour NN, Rogers L, Reibman J, Chupp GL. Characterization of Asthma Subgroups Associated with Circulating YKL-40 Levels. Eur Respir J 2017;4(50). [doi: 10.1183/13993003.00800-2017]

13. Gupta S, Siddiqui S, Haldar P, Entwisle JJ, Mawby D, Wardlaw AJ, Bradding P, Pavord ID, Green RH, Brightling CE. Quantitative analysis of high-resolution computed tomography scans in severe asthma subphenotypes. Thorax 2010;65(9):775–781. [doi: 10.1136/thx.2010.136374]

14. Haldar P, Pavord ID, Shaw DE, Berry MA, Thomas M, Brightling CE, Wardlaw AJ, Green RH. Cluster analysis and clinical asthma phenotypes. Am J Respir Crit Care Med 2008;178(3):218–224. PMID:18480428

15. Howrylak JA, Fuhlbrigge AL, Strunk RC, Zeiger RS, Weiss ST, Raby BA. Classification of childhood asthma phenotypes and long-term clinical responses to inhaled anti-inflammatory medications. J Allergy Clin Immunol [Internet] Elsevier Ltd; 2014;133(5):1289-1300.e12. PMID:24892144

16. Hsiao HP, Lin MC, Wu CC, Wang CC, Wang TN. Sex-Specific Asthma Phenotypes, Inflammatory Patterns, and Asthma Control in a Cluster Analysis. J Allergy Clin Immunol Pract [Internet] Elsevier Inc; 2019;7(2):556-567.e15. [doi: 10.1016/j.jaip.2018.08.008]

17. Ilmarinen P, Tuomisto LE, Niemelä O, Tommola M, Haanpää J, Kankaanranta H. Cluster Analysis on Longitudinal Data of Patients with Adult-Onset Asthma. J Allergy Clin Immunol Pract 2017;5(4):967-978.e3. PMID:28389304

18. Jang AS, Kwon HS, Cho YS, Bae YJ, Kim TB, Park JS, Park SW, Uh ST, Choi JS, Kim YH, Hwang HK, Moon HB, Park CS. Identification of subtypes of refractory asthma in Korean patients by cluster analysis. Lung 2013;191(1):87–93. PMID:23143671

19. Just J, Gouvis-Echraghi R, Rouve S, Wanin S, Moreau D, Annesi-Maesano I. Two novel, severe asthma phenotypes identified during childhood using a clustering approach. Eur Respir J 2012;40(1):55–60. PMID:22267763

20. Just J, Gouvis-Echraghi R, Couderc R, Guillemot-Lambert N, Saint-Pierre P. Novel severe wheezy young children phenotypes: Boys atopic multiple-trigger and girls nonatopic uncontrolled wheeze. J Allergy Clin Immunol [Internet] Elsevier Ltd; 2012;130(1):103-110.e8. PMID:22502798

21. Just J, Saint-Pierre P, Gouvis-Echraghi R, Boutin B, Panayotopoulos V, Chebahi N, Ousidhoum-Zidi A, Khau CA. Wheeze phenotypes in young children have different courses during the preschool period. Ann Allergy, Asthma Immunol [Internet] American College of Allergy, Asthma & Immunology; 2013;111(4):256–261. PMID:24054360

22. Just J, Saint-Pierre P, Gouvis-Echraghi R, Laoudi Y, Roufai L, Momas I, Annesi Maesano I. Childhood allergic asthma is not a single phenotype. J Pediatr [Internet] Elsevier Ltd; 2014;164(4):815–820. [doi: 10.1016/j.jpeds.2013.11.037]

23. Kaneko Y, Masuko H, Sakamoto T, Iijima H, Naito T, Yatagai Y, Yamada H, Konno S, Nishimura M, Noguchi E, Hizawa N. Asthma Phenotypes in Japanese Adults - Their Associations with the CCL5 ADRB2 Genotypes. Allergol Int [Internet] Elsevier Masson SAS; 2013;62(1):113–121. PMID:23267209

24. Khusial RJ, Sont JK, Loijmans RJB, Snoeck-Stroband JB, Assendelft PJJ, Schermer TRJ, Honkoop PJ. Longitudinal outcomes of different asthma phenotypes in primary care, an observational study. npj Prim Care Respir Med [Internet] Springer US; 2017;27(1):55. PMID:28974677

25. Kim TB, Jang AS, Kwon HS, Park JS, Chang YS, Cho SH, Choi BW, Park JW, Nam DH, Yoon HJ, Cho YJ, Moon HB, Cho YS, Park CS. Identification of asthma clusters in two independent Korean adult asthma cohorts. Eur Respir J 2013;41(6):1308–1314. PMID:23060627

26. Kim MA, Shin SW, Park JS, Uh ST, Chang HS, Bae DJ, Cho YS, Park HS, Yoon HJ, Choi BW, Kim YH, Park CS. Clinical characteristics of exacerbation-prone adult asthmatics identified by cluster analysis. Allergy, Asthma Immunol Res 2017;9(6):483–490. [doi: 10.4168/aair.2017.9.6.483]

27. Konno S, Taniguchi N, Makita H, Nakamaru Y, Shimizu K, Shijubo N, Fuke S, Takeyabu K, Oguri M, Kimura H, Maeda Y, Suzuki M, Nagai K, Ito YM, Wenzel SE, Nishimura M, HiCARAT Investigators. Distinct phenotypes of smokers with fixed airflow limitation identified by cluster analysis of severe asthma. Ann Am Thorac Soc 2018;15(1):33–41. [doi: 10.1513/AnnalsATS.201701-065OC]

28. Konstantellou E, Papaioannou AI, Loukides S, Patentalakis G, Papaporfyriou A, Hillas G, Papiris S, Koulouris N, Bakakos P, Kostikas K. Persistent airflow obstruction in patients with asthma : Characteristics of a distinct clinical phenotype. Respir Med [Internet] Elsevier Ltd; 2015;109(11):1404–1409. [doi: 10.1016/j.rmed.2015.09.009]

29. Labor M, Labor S, Jurić I, Fijačko V, Grle SP, Plavec D. Mood disorders in adult asthma phenotypes. J Asthma [Internet] 2018;55(1):57–65. [doi: 10.1080/02770903.2017.1306546]

30. Lavoie-Charland É, Bérubé J-C, Laviolette M, Boulet L-P, Bossé Y. Multivariate Asthma Phenotypes in Adults: The Quebec City Case-Control Asthma Cohort. Open J Respir Dis 2013;2013(November):133–142.

31. Lee HY, Ye YM, Kim SH, Ban GY, Kim SC, Kim JH, Shin YS, Park HS. Identification of phenotypic clusters of nonsteroidal anti-inflammatory drugs exacerbated respiratory disease. Allergy Eur J Allergy Clin Immunol 2017;72(4):616–626. [doi: 10.1111/all.13075]

32. Lefaudeux D, De Meulder B, Loza MJ, Peffer N, Rowe A, Baribaud F, Bansal AT, Lutter R, Sousa AR, Corfield J, Pandis I, Bakke PS, Caruso M, Chanez P, Dahlén SE, Fleming LJ, Fowler SJ, Horvath I, Krug N, Montuschi P, Sanak M, Sandstrom T, Shaw DE, Singer F, Sterk PJ, Roberts G, Adcock IM, Djukanovic R, Auffray C, Chung KF, U-BIOPRED Study Group. U-BIOPRED clinical adult asthma clusters linked to a subset of sputum omics. J Allergy Clin Immunol 2017;139(6):1797–1807. PMID:27773852

33. Lemiere C, Nguyen S, Sava F, D’Alpaos V, Huaux F, Vandenplas O. Occupational asthma phenotypes identified by increased fractional exhaled nitric oxide after exposure to causal agents. J Allergy Clin Immunol 2014;134(5):1063–1067. PMID:25262466

34. Loureiro CC, Sa-Couto P, Todo-Bom A, Bousquet J. Cluster analysis in phenotyping a Portuguese population. Rev Port Pneumol (English Ed [Internet] Sociedade Portuguesa de Pneumologia; 2015;21(6):299–306. PMID:26344641

35. Loza MJ, Djukanovic R, Chung KF, Horowitz D, Ma K, Branigan P, Barnathan ES, Susulic VS, Silkoff PE, Sterk PJ, Baribaud F, ADEPT (Airways Disease Endotyping for Personalized Therapeutics) Investigators, U-BIOPRED (Unbiased Biomarkers for the Prediction of Respiratory Disease Outcome Consortium) investigators. Validated and longitudinally stable asthma phenotypes based on cluster analysis of the ADEPT study. Respir Res [Internet] Respiratory Research; 2016;17(1):1–21. PMID:27978840

36. Mahut B, Peyrard S, Delclaux C. Exhaled nitric oxide and clinical phenotypes of childhood asthma. Respir Res 2011;12(65):1–8.

37. Meyer N, Nuss SJ, Siebenhüner A, Akdis CA, Menz G, Rothe T. Differential serum protein markers and the clinical severity of asthma. J Asthma Allergy 2014;67. [doi: 10.2147/jaa.s53920]

38. Meyer N, Dallinga JW, Nuss SJ, Moonen EJC, van Berkel JJBN, Akdis C, van Schooten FJ, Menz G. Defining adult asthma endotypes by clinical features and patterns of volatile organic compounds in exhaled air. Respir Res 2014;15(1):1–9. PMID:25431084

39. Moore WC, Meyers DA, Wenzel SE, Teague WG, Li H, Li X, D’Agostino R, Castro M, Curran-Everett D, Fitzpatrick AM, Gaston B, Jarjour NN, Sorkness R, Calhoun WJ, Chung KF, Comhair SAA, Dweik RA, Israel E, Peters SP, Busse WW, Erzurum SC, Bleecker ER. Identification of asthma phenotypes using cluster analysis in the severe asthma research program. Am J Respir Crit Care Med 2010;181(4):315–323. PMID:19892860

40. Moore WC, Hastie AT, Li X, Li H, Busse WW, Jarjour NN, Wenzel SE, Peters SP, Meyers DA, Bleecker ER. Sputum neutrophil counts are associated with more severe asthma phenotypes using cluster analysis. J Allergy Clin Immunol 2014;133(6). PMID:24332216

41. Newby C, Heaney LG, Menzies-Gow A, Niven RM, Mansur A, Bucknall C, Chaudhuri R, Thompson J, Burton P, Brightling C. Statistical cluster analysis of the british thoracic society severe refractory asthma registry: Clinical outcomes and phenotype stability. PLoS One 2014;9(7). PMID:25058007

42. Park HW, Song WJ, Kim SH, Park HK, Kim SH, Kwon YE, Kwon HS, Kim TB, Chang YS, Cho YS, Lee BJ, Jee YK, Jang AS, Nahm DH, Park JW, Yoon HJ, Cho YJ, Choi BW, Moon HB, Cho SH. Classification and implementation of asthma phenotypes in elderly patients. Ann Allergy, Asthma Immunol [Internet] American College of Allergy, Asthma & Immunology; 2015;114(1):18–22. PMID:25455518

43. Patrawalla P, Kazeros A, Rogers L, Shao Y, Liu M, Fernandez-Beros ME, Shang S, Reibman J. Application of the Asthma Phenotype Algorithm from the Severe Asthma Research Program to an Urban Population. PLoS One 2012;7(9). PMID:23028556

44. Pérez-Losada M, Authelet KJ, Hoptay CE, Kwak C, Crandall KA, Freishtat RJ. Pediatric asthma comprises different phenotypic clusters with unique nasal microbiotas. Microbiome Microbiome; 2018;6(1):1–13. [doi: 10.1186/s40168-018-0564-7]

45. Qiu R, Xie J, Chung KF, Li N, Yang Z, He M, Li J, Chen R, Zhong N, Zhang Q. Asthma Phenotypes Defined From Parameters Obtained During Recovery From a Hospital-Treated Exacerbation. J Allergy Clin Immunol Pract [Internet] Elsevier Inc; 2018;6(6):1960–1967. [doi: 10.1016/j.jaip.2018.02.012]

46. Ruggieri S, Drago G, Longo V, Colombo P, Balzan M, Bilocca D, Zammit C, Montefort S, Scaccianoce G, Cuttitta G, Viegi G, Cibella F, Borg C, Bucchieri S, Ferrante G, L’Abbate L, La Grutta S, Melis MR, Rizzo G, Minardi R, Piva G, Ristagno R. Sensitization to dust mite defines different phenotypes of asthma: A multicenter study. Pediatr Allergy Immunol 2017;28(7):675–682. PMID:28783215

47. Sakagami T, Hasegawa T, Koya T, Furukawa T, Kawakami H, Kimura Y, Hoshino Y, Sakamoto H, Shima K, Kagamu H, Suzuki EI, Narita I. Cluster analysis identifies characteristic phenotypes of asthma with accelerated lung function decline. J Asthma 2014;51(2):113–118. PMID:24102534

48. Schatz M, Hsu JWY, Zeiger RS, Chen W, Dorenbaum A, Chipps BE, Haselkorn T. Phenotypes determined by cluster analysis in severe or difficult-to-treat asthma. J Allergy Clin Immunol [Internet] Elsevier Ltd; 2014;133(6):1549–1556. PMID:24315502

49. Seino Y, Hasegawa T, Koya T, Sakagami T, Mashima I, Shimizu N, Muramatsu Y, Muramatsu K, Suzuki E, Kikuchi T, Niigata Respiratory Disease Study Group. A Cluster Analysis of Bronchial Asthma Patients with Depressive Symptoms. Intern Med 2018;57:1967–1975. PMID:29526967

50. Sekiya K, Nakatani E, Fukutomi Y, Kaneda H, Iikura M, Yoshida M, Takahashi K, Tomii K, Nishikawa M, Kaneko N, Sugino Y, Shinkai M, Ueda T, Tanikawa Y, Shirai T, Hirabayashi M, Aoki T, Kato T, Iizuka K, Homma S, Taniguchi M, Tanaka H. Severe or life-threatening asthma exacerbation: patient heterogeneity identified by cluster analysis. Clin Exp Allergy 2016;46(8):1043–1055. PMID:27041475

51. Sendín-Hernández MP, Ávila-Zarza C, Sanz C, García-Sánchez A, Marcos-Vadillo E, Muñoz-Bellido FJ, Laffond E, Domingo C, Isidoro-García M, Dávila I. Cluster Analysis Identifies 3 Phenotypes within Allergic Asthma. J Allergy Clin Immunol Pract 2018;6(3):955-961.e1. [doi: 10.1016/j.jaip.2017.10.006]

52. Serrano-Pariente J, Rodrigo G, Fiz JA, Crespo A, Plaza V. Identification and characterization of near-fatal asthma phenotypes by cluster analysis. Allergy Eur J Allergy Clin Immunol 2015;70(9):1139–1147. PMID:26011771

53. Su MW, Lin WC, Tsai CH, Chiang BL, Yang YH, Lin YT, Wang LC, Lee JH, Chou CC, Wu YF, Yeh YL, Lee YL. Childhood asthma clusters reveal neutrophil-predominant phenotype with distinct gene expression. Allergy Eur J Allergy Clin Immunol 2018;73(10):2024–2032. [doi: 10.1111/all.13439]

54. Sutherland ER, Goleva E, King TS, Lehman E, Stevens AD, Jackson LP, Stream AR, Fahy J V., Leung DYM. Cluster analysis of obesity and asthma phenotypes. PLoS One 2012;7(5):1–7. PMID:22606276

55. Tsukioka K, Koya T, Ueno H, Hayashi M, Sakagami T, Hasegawa T, Arakawa M, Suzuki E, Kikuchi T. Phenotypic analysis of asthma in Japanese athletes. Allergol Int [Internet] Elsevier B.V; 2017;66(4):550–556. [doi: 10.1016/j.alit.2017.02.009]

56. Ueno H, Koya T, Hasegawa T, Hayashi M, Yoshizawa L, Suzuki E, Kikuchi T. A study of factors related to asthma exacerbation using a questionnaire survey in Niigata Prefecture, Japan. Asian Pacific J Allergy Immunol 2019; [doi: 10.12932/ap-080918-0404]

57. Wang L, Liang R, Zhou T, Zheng J, Liang BM, Zhang HP, Luo FM, Gibson PG, Wang G. Identification and validation of asthma phenotypes in Chinese population using cluster analysis. Ann Allergy, Asthma Immunol [Internet] American College of Allergy, Asthma & Immunology; 2017;119(4):324–332. PMID:28866310

58. Wu W, Bleecker E, Moore W, Busse WW, Castro M, Chung KF, Calhoun WJ, Erzurum S, Gaston B, Israel E, Curran-Everett D, Wenzel SE. Unsupervised phenotyping of Severe Asthma Research Program participants using expanded lung data. J Allergy Clin Immunol [Internet] Elsevier Ltd; 2014;133(5):1280–1288. PMID:24589344

59. Wu W, Bang S, Bleecker ER, Castro M, Denlinger L, Erzurum SC, Fahy J V, Fitzpatrick AM, Gaston BM, Hastie AT, Israel E, Jarjour NN, Levy BD, Mauger DT, Meyers DA, Moore WC, Peters M, Phillips BR, Phipatanakul W, Sorkness RL, Wenzel SE. Multiview Cluster Analysis Identifies Variable Corticosteroid Response Phenotypes in Severe Asthma. Am J Respir Crit Care Med [Internet] 2019;199(11):rccm.201808-1543OC. [doi: 10.1164/rccm.201808-1543OC]

60. Ye WJ, Xu WG, Guo XJ, Han FF, Peng J, Li XM, Guan W Bin, Yu LW, Sun JY, Cui ZL, Song L, Zhang Y, Wang YM, Yang TY, Ge XH, Yao D, Liu S. Differences in airway remodeling and airway inflammation among moderate-severe asthma clinical phenotypes. J Thorac Dis 2017;9(9):2904–2914. PMID:29221262

61. Youroukova VM, Dimitrova DG, Valerieva AD, Lesichkova SS, Velikova T V., Ivanova-Todorova EI, Tumangelova-Yuzeir KD. Phenotypes Determined by Cluster Analysis in Moderate to Severe Bronchial Asthma. Folia Med (Plovdiv) [Internet] 2017;59(2):165–173. [doi: 10.1515/folmed-2017-0031]

62. Zaihra T, Walsh CJ, Ahmed S, Fugère C, Hamid QA, Olivenstein R, Martin JG, Benedetti A. Phenotyping of difficult asthma using longitudinal physiological and biomarker measurements reveals significant differences in stability between clusters. BMC Pulm Med [Internet] BMC Pulmonary Medicine; 2016;16(1):1–8. PMID:27165150

63. Zoratti EM, Krouse RZ, Babineau DC, Pongracic JA, O’Connor GT, Wood RA, Khurana Hershey GK, Kercsmar CM, Gruchalla RS, Kattan M, Teach SJ, Sigelman SM, Gergen PJ, Togias A, Visness CM, Busse WW, Liu AH. Asthma phenotypes in inner-city children. J Allergy Clin Immunol [Internet] Elsevier Inc.; 2016;138(4):1016–1029. PMID:27720016
