# Supplementary material for: Challenges of Clustering Multimodal Clinical Data: Review of Applications in Asthma Subtyping
Source: JMIR Med Inform. 2020 May 28;8(5):e16452. doi: 10.2196/16452 (PMC7290450; doi:10.2196/16452)
Supplement: Multimedia Appendix 4 [file medinform_v8i5e16452_app4.docx]

Multimedia Appendix 4

Breakdown of methods used by the 11 studies that did not use the three most common clustering methods.

| **Clustering method** | **Data type** | **Dissimilarity** | **Scaling of continuous features** | **Categorical features encoded as binary?** | **n (%)^a^** |
| --- | --- | --- | --- | --- | --- |
| Hierarchical clustering (linkage unspecified)  N = 2 (3%) | Continuous | Euclidean assumed | No details | **-** | 1 (50) |
|  |  | Spearman's rho | z-scores | **-** | 1 (50) |
| k-medoids  N = 2 (3%) | Continuous | Euclidean stated | Centre-scaled | **-** | 1 (50) |
|  | Mixed | Gower^b^ | Scaled but method unspecified | No | 1 (50) |
| Fuzzy k-medoids  N = 1 (2%) | Continuous | Euclidean stated | Average absolute deviation | **-** | 1 (100) |
| Hierarchical with average linkage  N = 1 (2%) | Continuous | Euclidean stated | z-scores for one feature | **-** | 1 (100) |
| k-means to pre-cluster then hierarchical with Ward's linkage  N = 1 (2%) | Mixed | Euclidean assumed | Scaled but method unspecified | No | 1 (100) |
| Multiple kernel k-means clustering  N = 1 (2%) | Mixed | Euclidean assumed | z-scores | Yes | 1 (100) |
| Pre-cluster step followed by hierarchical clustering  N = 1 (2%) | Mixed | Euclidean assumed | No details | No | 1 (100) |
| Spectral clustering  N = 1 (2%) | Mixed | Euclidean assumed | Scaled to unit vectors | Yes | 1 (100) |
| Unclear  N = 1 (2%) | Mixed | Log-likelihood stated | z-scores | Yes | 1 (100) |

^a^ % calculation grouped by cluster analysis method
^b^ Computing the Gower coefficient normalizes the distance between feature samples by dividing by the feature range. Therefore, it is not necessary to normalize continuous features prior to computing the Gower coefficient.
