# Supplementary material for: Challenges of Clustering Multimodal Clinical Data: Review of Applications in Asthma Subtyping
Source: JMIR Med Inform. 2020 May 28;8(5):e16452. doi: 10.2196/16452 (PMC7290450; doi:10.2196/16452)
Supplement: Multimedia Appendix 5 [file medinform_v8i5e16452_app5.docx]

Multimedia Appendix 5

An illustrative example of why Gower’s coefficient should not be used with hierarchical clustering and Ward’s linkage:

<https://github.com/elsie-h/ward_gower/blob/master/writeup.md>
